# Supplementary material for: Venomix: a simple bioinformatic pipeline for identifying and characterizing toxin gene candidates from transcriptomic data
Source: PeerJ. 2018 Jul 31;6:e5361. doi: 10.7717/peerj.5361 (PMC6074769; doi:10.7717/peerj.5361)
Supplement: Supplemental Information 6 [file peerj-06-5361-s006.gz › FinalOutput_1E-6/C-type_lectin_mannose-binding_isoform_30/finaltree.pdf]

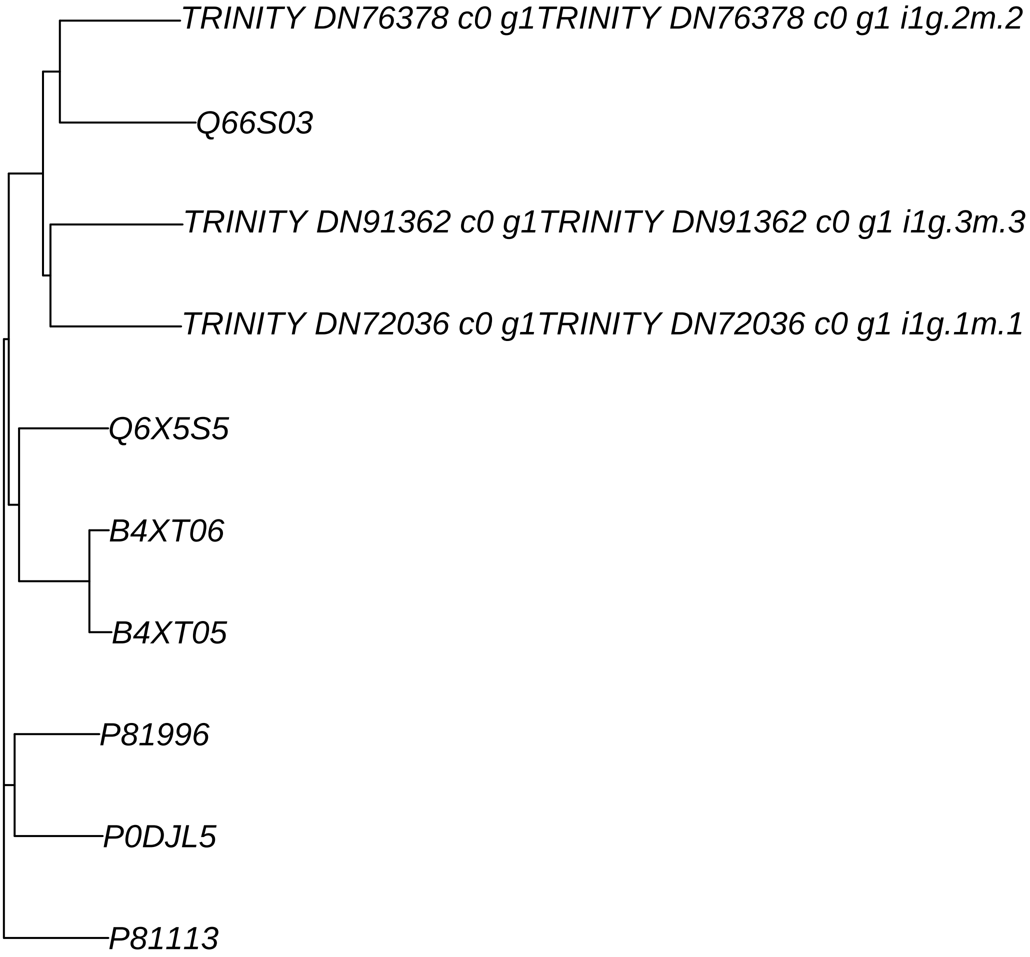
TRINITY DN76378 c0 g1TRINITY DN76378 c0 g1 i1g.2m.2
Q66S03
TRINITY DN91362 c0 g1TRINITY DN91362 c0 g1 i1g.3m.3
TRINITY DN72036 c0 g1TRINITY DN72036 c0 g1 i1g.1m.1
Q6X5S5
B4XT06
B4XT05
P81996
P0DJL5
P81113
